# High spatiotemporal resolution hyperpolarized ^13^C angiography

**DOI:** 10.1186/1532-429X-18-S1-Q30

**Published:** 2016-01-27

**Authors:** Galen D Reed, Cornelius von Morze, Nii O Addy, R Reeve Ingle, Kenneth O Johnson, William R Overall, Bob S Hu, Daniel B Vigneron, Peder E Larson, Juan M Santos

**Affiliations:** 1 grid.420669.fHeartVista, San Francisco, CA USA; 2 grid.266102.10000000122976811Radiology and Biomedical Imaging, University of California San Francisco, San Francisco, CA USA; 3 grid.416759.80000000404603124Cardiology, Palo Alto Medical Foundation, Palo Alto, CA USA

## Background

Sub-millimeter resolution, background-free magnetic resonance angiography (MRA) has been performed previously using dynamic nuclear polarization (DNP)- enhanced ^13^C labeled small molecules [1-4]. This approach of contrast-enhanced MRA is appealing since many of the commonly used DNP substrates are endogenous and could be potentially used in large doses in patients with renal insufficiency. The aim of this study was to combine high spatial resolution MRA with a high frame rate spiral readout [5], and to test the feasibility of ^13^C magnetic resonance fluoroscopy in rats on a clinical imaging system.

## Methods

Male Sprague Dawley rats were anesthetized with a 1% isofluorane / O2 mixture and placed in a dual-tuned 1H/13C birdcage transceiver inside a clinical GE 3T scanner. [^13^C,^15^N]urea was polarized on an Oxford Instruments HyperSense, dissolved in a saline / phosphate buffer solution, and administered via lateral tail vein catheters.

Real time scan control and image reconstruction was implemented on the RTHawk platform [6]. An SSFP sequence was tailored for transient phase hyperpolarized imaging by using a 10-step Kaiser-Bessel flip angle ramp with a terminal flip angle of 180 degrees. A variable density spiral readout (12 cm FOV at k = 0, 8 cm FOV at kmax, 1 mm resolution) was utilized for the maximization of signal detection time (8 ms) within a TR (13 ms). The 30 interleaves were gridded, density compensated, and a single image was reconstructed for every 2 TRs giving an effective 26 ms frame rate. Images were acquired as coronal and axial projections.

## Results

Figure 2a shows axial projections initiated 20 s after the beginning of injection. Although the aorta, vena cava, and left and right ventricles of the heart can be depicted, cardiac motion could not be clearly resolved. Coronal images (Figure 2b) acquired with a lower terminal flip angle (90 degrees) showed some image shading over the kidneys likely due to banding. Several aorta branches could be detected including the renal and possibly the hepatic arteries. Contrast agent flow through the latter can be seen in the 1st through 4th images. The dynamic signal intensity of a line profile (yellow trace) is shown in Figure 2c. A periodic 1 mm displacement of the aorta was due to respiratory motion.

**Figure 1:**
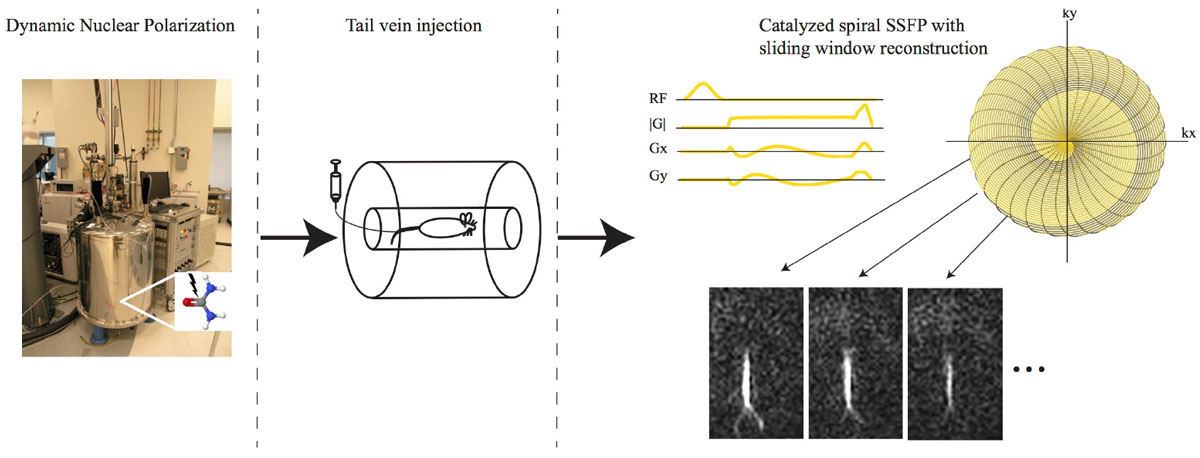
**Schematic of the experiment**.

**Figure 2:**
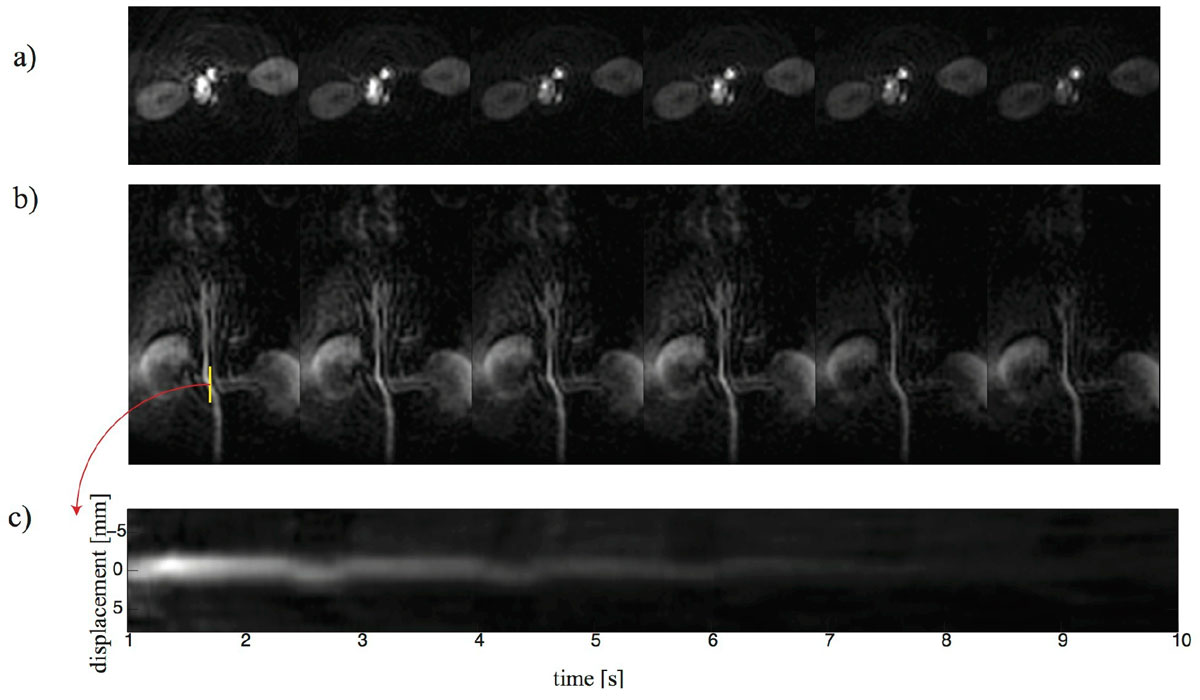
**a) axial, and b) coronal projection angiograms**. c) a dynamic line profile (yellow trace) showing aorta displacement during respiratory motion.

## Conclusions

This study showed preliminary results of high spatial (~1 mm) and high temporal (~30 ms) resolution acquisition of hyperpolarized ^13^C substrates. Real time ^13^C angiography could address the potential risks of radiation exposure from X-ray fluoroscopy and nephrotoxicity inherent to most common MRI and CT contrast agents. Image quality will likely improve with the use of multi-frequency reconstruction, parallel imaging, and flow-refocusing gradient pulses.
